# Risk factors and surrogate indicators for cardiovascular disease are prevalent in Common Variable Immunodeficiency and associate with inflammatory phenotype

**DOI:** 10.3389/fimmu.2026.1756049

**Published:** 2026-03-02

**Authors:** Aidan Jia Sheng Yu, Fernando Moreira, Andrew Symes, Keegan Curlewis, Mary O’Sullivan, Joseph Jayasundera, Fatema-Zahra El Rhermoul, Charley Lever, Kostadin Stoenchev, Ke Li Chow, Omer Faruk Uysal, Ahmad M Alharbi, Sarita Workman, Neil Halliday, Arian Laurence, Nisha Verma, Susan Tadros, Sorena Kiani-Alikhan, Joseph Barnett, Siobhan O Burns, John R Hurst, David M Lowe

**Affiliations:** 1 Department of Clinical Immunology, Royal Free London NHS Foundation Trust, London, United Kingdom; 2 Department of Radiology, Royal Free Hospital London NHS Foundation Trust, London, United Kingdom; 3 UCL Respiratory, University College London, London, United Kingdom; 4 Sheila Sherlock Liver Centre, Royal Free London NHS Foundation Trust, London, United Kingdom; 5 University College London Institute for Liver and Digestive Health, University College London, London, United Kingdom; 6 Institute of Immunity and Transplantation, University College London, London, United Kingdom

**Keywords:** atherosclerosis, cardiovascular risk, common variable immunodeficiency, coronary artery calcification, d-dimer, endothelial dysfunction, inflammatory phenotype, von Willebrand factor

## Abstract

**Background:**

Common variable immunodeficiency (CVID) is traditionally characterised by recurrent infections and immune dysregulation, but growing evidence suggests an increased risk of endothelial dysfunction and premature atherosclerosis in this population.

**Objective:**

To evaluate cardiovascular risk in patients with CVID through integration of clinical risk factors, biomarkers of endothelial dysfunction, and radiographic surrogates of subclinical atherosclerosis.

**Methods:**

A total of 101 CVID patients and 56 matched household controls were recruited. Data collected included cardiovascular risk factors, blood biomarkers (D-dimer, von Willebrand factor [vWF], fibrinogen, ESR, CRP), and immunological profiles. Existing imaging was reviewed, including thoracic CT for assessment of coronary artery calcification (CAC) and FibroScan for controlled attenuation parameter (CAP) scores; aortic pulse wave velocity (aPWV) was measured in a subset of participants. Subgroup analysis compared infection-only versus inflammatory/complex phenotypes of CVID.

**Results:**

CVID patients demonstrated high rates of hyperlipidaemia (38.6%), hypertension (23.8%), and diabetes/prediabetes (14.9%). CAC was present in 37%, with 82.4% having no known prior cardiovascular disease. Hepatic steatosis and elevated aPWV were observed in 30% and 6.5%, respectively. Patients with CAC were older and had higher rates of hypertension, diabetes, hyperlipidaemia, chronic kidney disease, elevated median vWF (227.5 vs 167 IU/dL, p=0.001), D-dimer (370.5 vs 271 ng/mL, p=0.011), and aPWV (8.2 vs 6.0 m/s, p=0.006). Patients with an inflammatory phenotype had higher vWF (224 vs 163 IU/dL, p<0.001) and D-dimer (314 vs 205 ng/mL, p=0.043) levels than those with infection-only CVID.

**Conclusion:**

CVID is associated with a substantial burden of cardiovascular risk factors and subclinical atherosclerosis, especially in the inflammatory phenotype.

## Introduction

Chronic inflammatory conditions such as HIV, rheumatological, and autoimmune diseases are associated with endothelial dysfunction, accelerated atherosclerosis, and premature cardiovascular or cerebrovascular disease (1, 2). Common variable immunodeficiency (CVID), a primary immunodeficiency characterised by reduced serum IgG, low IgA and/or IgM, has most commonly been associated with recurrent infections (3, 4). Additionally, patients with CVID may develop a range of inflammatory complications, including granulomatous lymphocytic interstitial lung disease (GLILD), enteropathy, lymphadenopathy, autoimmune cytopenia, arthritis, granulomatous inflammation and CVID-associated liver disease (5). There is now growing recognition of non-infectious complications in CVID, including cardiovascular involvement, especially as infectious complications are now better managed through optimised immunoglobulin replacement and antibiotic therapy (6–9).

Emerging data suggest that CVID patients may be predisposed to endothelial dysfunction and increased rates of cardiovascular and cerebrovascular events (10). In a cohort of 83 CVID patients, the risk of coronary heart disease and peripheral vascular disease was significantly elevated compared to matched controls (odds ratios 2.4 and 12.5, respectively), with an overall cardiovascular disease incidence of 21.7% versus 9.6% (6). Other studies have reported cardiovascular disease in primary immunodeficiency patients, with heart failure occurring in 10% versus 4% in the general population (11), hypertension in 25.5% (12) and 18% (8), and cardiac disease in 9% across various cohorts (13). However, many of these findings come from small cohorts or registry analyses which are subject to reporting bias.

CVID is often marked by persistent systemic inflammation, with elevated levels of C-reactive protein (CRP), soluble CD25, TNF, and soluble CD14 (8, 9, 14). Multiple studies have also reported lipid abnormalities in CVID, including elevated oxidised low-density lipoprotein (LDL), reduced high-density lipoprotein (HDL), and decreased Apolipoprotein-A1 (8, 9, 12, 15).

Increased arterial stiffness, a surrogate marker of cardiovascular risk, has been observed in CVID patients, particularly those with metabolic syndrome (7). Certain subgroups may carry a higher risk, for example patients with non-infectious complications have significantly lower HDL compared to those with an infection-only phenotype (8). Other immune abnormalities, such as lymphoproliferation, splenomegaly and reduced CD8+ T or B cell counts, are more prevalent in CVID patients with cardiac disease, suggesting that inflammation-prone phenotypes may be especially vulnerable to cardiovascular disease (13).

Traditional cardiovascular risk factors, including smoking history, hypertension, and impaired glucose tolerance, appear common but are not routinely recorded in many centres (12). Despite the growing evidence, major cardiovascular events such as myocardial infarction and stroke remain infrequently reported in CVID, and definitive risk remains uncertain. If CVID contributes to premature atherosclerosis, understanding and mitigating disease-specific cardiovascular risks will be critical. This may warrant integrating management strategies targeting modifiable risks, such as dyslipidaemia, hypertension, glucose intolerance, diet, and exercise. These strategies are well established in HIV care but are not yet routinely applied in immunology practice (16).

## Methods

This study was a single-centre, observational cohort study with prospective recruitment and retrospective review of imaging and laboratory data.

### Patient cohort

All patients with a diagnosis of CVID were identified using the Electronic Patient Record (EPR). Patients were recruited in the outpatient setting. CVID was diagnosed by a consultant immunologist according to internationally accepted guidelines (3), typically presenting with recurrent infections, hypogammaglobulinemia, and failure to respond to immunisation. Patients aged ≥18 years meeting these criteria were eligible for inclusion. Exclusion criteria included secondary hypogammaglobulinemia and an inability to provide informed consent. Patients with incomplete laboratory data or CT imaging were excluded from the relevant analyses.

Patients were asked to complete questionnaires that collected demographic information (age, sex, ethnicity) and cardiovascular risk factors, including hypertension, hyperlipidaemia, diabetes mellitus, chronic kidney disease (estimated glomerular filtration rate <60 mL/min/1.73 m²), smoking history, and family history of coronary artery disease. The reported cases of hypertension, hyperlipidaemia, and diabetes mellitus include both self-reported diagnoses and newly identified cases based on elevated blood pressure, glycated haemoglobin (HbA1c), or cholesterol levels detected during testing. Hypertension was defined as a systolic blood pressure ≥140 mmHg or diastolic blood pressure ≥90 mmHg, in accordance with guidelines from the European Society of Cardiology (ESC) and the World Health Organization (WHO) (17, 18). Hyperlipidaemia was defined as a total cholesterol level >5.0 mmol/L, and diabetes or prediabetes was defined as an HbA1c level >42 mmol/mol, based on the local laboratory reference range. Cardiovascular disease, including coronary artery disease (CAD), cerebrovascular accidents (CVA), and peripheral vascular disease (PVD) were self-reported. All information was subsequently cross-validated through the EPR.

The same questionnaires were also distributed to household members of patients, who served as a control group. This group was selected to best match the patient cohort in terms of age and lifestyle factors. Controls were eligible for inclusion if they were aged ≥18 years, able to provide informed consent, and had no known diagnosis of primary or secondary immunodeficiency.

### Blood collection

Blood samples were collected either in the outpatient clinic or immediately before patients received their regular intravenous immunoglobulin replacement therapy. Blood samples were analysed to evaluate renal function, lipid profiles, and HbA1c levels, along with markers of inflammation (CRP and erythrocyte sedimentation rate [ESR]). Markers of coagulability, including fibrinogen, D-dimer, von Willebrand factor (vWF), plasma viscosity, and lupus anticoagulant were also measured. Immunological tests included measurement of switched memory B cells, CD21^low^ B cells (more than 10%) (19), CD19 cell counts, and serum levels of IgA, and IgM. All laboratory analyses were performed at the pathology department of the local tertiary hospital, using standardised protocols and accredited diagnostic platforms.

### Evaluation of surrogate markers of cardiovascular disease

Hepatic steatosis was evaluated using the Controlled Attenuation Parameter (CAP) obtained via liver elastography (FibroScan), where this had been done as part of routine care. CAP thresholds were based on a meta-analysis by Karlas et al., and defined as follows: normal (<248 dB/m), mild (248–267 dB/m), moderate (268–279 dB/m), and severe (≥280 dB/m) (20).

Arterial stiffness was evaluated by measuring aortic pulse wave velocity (aPWV) between the carotid and femoral arteries using Vicorder (Skidmore Medical) equipment. aPWV is an established marker of arterial stiffness (21, 22). The reference range varies with age. An aPWV of <6 m/s is considered normal in individuals under 30 years of age (21). European guidelines define a universal threshold of aPWV ≥10 m/s as indicative of increased arterial stiffness and cardiovascular risk, irrespective of age (23). Patients were also grouped into three aPWV categories: <6 m/s, 6–10 m/s, and ≥10 m/s.

Coronary artery calcification was assessed by a radiologist using non-gated thoracic CT scans performed as part of routine clinical care and categorised as none, mild, moderate, or severe, in accordance with guidance from the British Society of Cardiovascular Imaging/British Society of Cardiac Computed Tomography (BSCI/BSCCT) and the British Society of Thoracic Imaging (BSTI) (24). The reporting radiologist was blinded to all clinical and laboratory data, including patient group classification (infection-only vs inflammatory phenotype).

Both FibroScan (CAP) and thoracic CT imaging were performed as part of standard clinical evaluation and were obtained within a median of two years prior to analysis.

The patient cohort was stratified into two groups based on evidence of cardiovascular disease, defined by coronary artery calcification (mild or greater) on CT imaging versus no calcification. In addition, the cohort was divided into two groups based on clinical phenotype: infection only and inflammatory. Patients were classified as having an inflammatory phenotype if they had documented non-infectious complications, including GLILD, enteropathy, autoimmune cytopenia, arthritis, granulomatous disease, CVID-related liver disease, or other immune mediated manifestations.

### Statistical methods

Continuous variables were compared between groups using either Student’s t-test or the Mann-Whitney U test, depending on data distribution. The normality of data distribution was analysed using Shapiro-Wilk test. Categorical variables were analysed using Chi-square test. Correlation analysis was performed using non-parametric Spearman’s rank correlation. Statistical analyses were performed using GraphPad Prism (version 10.4.2) and IBM SPSS Statistics (version 30).

### Ethics

All patients provided written informed consent under NHS Research Ethics Committee approved protocols (04/Q0501/119 for patients and 08/H0720/46 for controls).

## Results

### Patient demographics

A total of 101 patients with CVID were included in the study (mean age: 52.1 years; range 18–83 years; 45.5% male [n=46], 54.5% female [n=55]). All patients completed the study questionnaires. Most patients (88.1%, n=89) identified as white. A total of 97% (n=98) were receiving immunoglobulin replacement therapy. Of these patients, 60.4% (n=61) were on intravenous immunoglobulin replacement and 36.6% (n=37) were on subcutaneous immunoglobulin replacement.

A control group of 56 individuals, consisting of household members of the patients, was also recruited (mean age: 51.6 years; 48.2% male [n=27], 51.8% female [n=29]). Among the control group, 91.1% (n=51) identified as white. Participant demographics are summarised in **Table 1**.

**Table 1:** Comparison of demographics, cardiovascular risk factors and cardiovascular disease in CVID patients and household control.

Variable	CVID (n= 101)	Control (n= 56)	*P* value
Age (years)	52.1 ± 15.9	51.6 ± 16.5	0.863^b^
Sex			
Male	46 (45.5%)	27 (48.2%)	0.748^a^
Female	55 (54.5%)	29 (51.8%)	
Ethnicity			
White	89 (88.1%)	51 (91.1%)	0.569^a^
Non-white	12 (11.9%)	5 (8.9%)	
Cardiovascular risk factors (self-reported or established via diagnostic tests)			
Hypertension	24 (23.8%)	14 (25%)	0.862^a^
Hyperlipidaemia	39 (38.6%)	13 (23.2%)	**0.050** ^a^
Diabetes/Prediabetes	15 (14.9%)	8 (14.3%)	0.924^a^
Chronic Kidney Disease	7 (6.9%)	0 (0%)	**0.044** ^a^
Smoking history	17 (16.8%)	5 (8.9%)	0.172^a^
Cardiovascular disease			
Coronary Artery Disease	5 (5.0%)	4 (7.1%)	0.571^a^
Cerebrovascular Accident	2 (2.0%)	3 (5.4%)	0.248^a^
Peripheral Vascular Disease	1 (1.0%)	1 (1.8%)	0.67^a^

a Chi-squared test

b Student’s t-test

CVID, common variable immunodeficiency disorderBold values indicate statistical significance (p < 0.05).

### Cardiovascular risk factors are common in CVID

Classical cardiovascular risk factors were common in those with CVID: a history of hyperlipidaemia was present in 38.6% (n=39), hypertension in 23.8% (n=24), and 14.9% (n=15) had either diabetes or elevated HbA1c suggestive of prediabetes. Chronic kidney disease (CKD) was identified in 6.9% (n=7). Self-reported hyperlipidaemia and CKD were more common in the CVID patients compared to controls (**Table 1**).

With respect to cardiovascular disease, 5% (n=5) had coronary artery disease (CAD), 2.0% (n=2) had a history of cerebrovascular accident (CVA), and 1.0% (n=1) were diagnosed with peripheral vascular disease (PVD). No statistically significant differences in cardiovascular disease were observed when compared to the control group (**Table 1**), including when the CVID cohort was restricted to those with matched household controls (**Supplementary Table 1**).

### Blood markers of coagulation and endothelial dysfunction are frequently elevated in CVID

Immunology and coagulation parameters were available for 86 patients (85.1%), as summarised in **Table 2**.

**Table 2:** Immunologic parameters and blood markers of endothelial dysfunction, cholesterol levels and blood pressure measurements.

Immunology Parameters	n	Median (IQR) or n (%)
IgA (g/L)	86	0.1 (0.1 – 0.1)
IgM (g/L)	86	0.1 (0.1 – 0.4)
IgG (g/L)	86	10.1 (8.2 – 11.7)
Switched Memory B cells (% of B cells)	72	2.66% (1.25 – 6.70)
CD21^low^ B-cells (% of B cells)	72	11.96% (6.64 – 27.29)
CD21^low^ B-cells >10% of B cells	–	44 (61.1%)
Blood markers of endothelial dysfunction		
Platelet count (x10^9^/L)	86	189.5 (128.8 – 239.5)
Fibrinogen (g/L)	86	3.35 (2.80 – 4.00)
Fibrinogen >4g/L	86	20 (23.3%)
D-dimer (ng/mL)	86	305 (190.0 – 454.3)
D-dimer >400 ng/mL	–	27 (31.4%)
Von Willebrand Factor (IU/dL)	86	198.5 (151.5 – 263.0)
Von Willebrand Factor > 175 IU/dL	–	49 (57%)
Plasma Viscosity (mPa)	86	1.63 (1.56 – 1.74)
Plasma Viscosity >1.75 mPa	–	18 (20.9%)
Positive Lupus Anticoagulant	85	6 (7%)
CRP (mg/L)	86	2.25 (1.00 – 5.00)
CRP >5 mg/L	–	22.1% (n=19)
ESR (mm/hr)	86	8 (2 – 15)
ESR >20mm/hr	–	51 (59.3%)
Cholesterol, HbA1c and blood pressure		Mean (± SD) or n (%)
Total cholesterol (mmol/L)	94	4.49 (± 1.08)
Total cholesterol > 5 mmol/L	–	26 (27.7%)
LDL (mmol/L)	92	2.44 (± 0.90)
LDL > 2.3 mmol/L	–	43 (46.7%)
HDL (mmol/L)	94	1.39 (± 0.52)
HDL < 1 mmol/L	–	23 (24.5%)
Triglycerides (mmol/L)	94	1.49 (± 0.83)
Triglycerides > 2.3 mmol/L	–	11 (11.7%)
Hba1C (mmol/mol)	92	36.1 (± 10.3)
Hba1C > 42 mmol/mol	–	10 (10.9%)
Systolic blood pressure (mmHg)	86	124 (± 17.3)
Diastolic blood pressure (mmHg)	86	75.7 (± 9.5)
Blood pressure ≥140/90 mmHg	–	11 (12.8%)

Immunoglobulin A, (IgA); immunoglobulin M, (IgM); immunoglobulin G, (IgG); CRP, C-reactive protein; ESR, erythrocyte sedimentation rate; low-density lipoprotein (LDL), high-density lipoprotein (HDL); Hba1C, Haemoglobin A1c

The median trough IgG level in the patient cohort was 10.1 g/L, median IgA level was 0.1 g/L, and the median IgM level was 0.1 g/L. 83 patients (96.5%) were on immunoglobulin replacement therapy. The median percentage of switched memory B cells was 2.66% of total B cells (reference range: 6.5-29.1%).

CD21^low^ B-cell data were available for 72 patients. Elevated CD21^low^ B-cells (>10% of B-cells) were observed in 61.1% (n=44) of the cohort.

**Figure 1:**
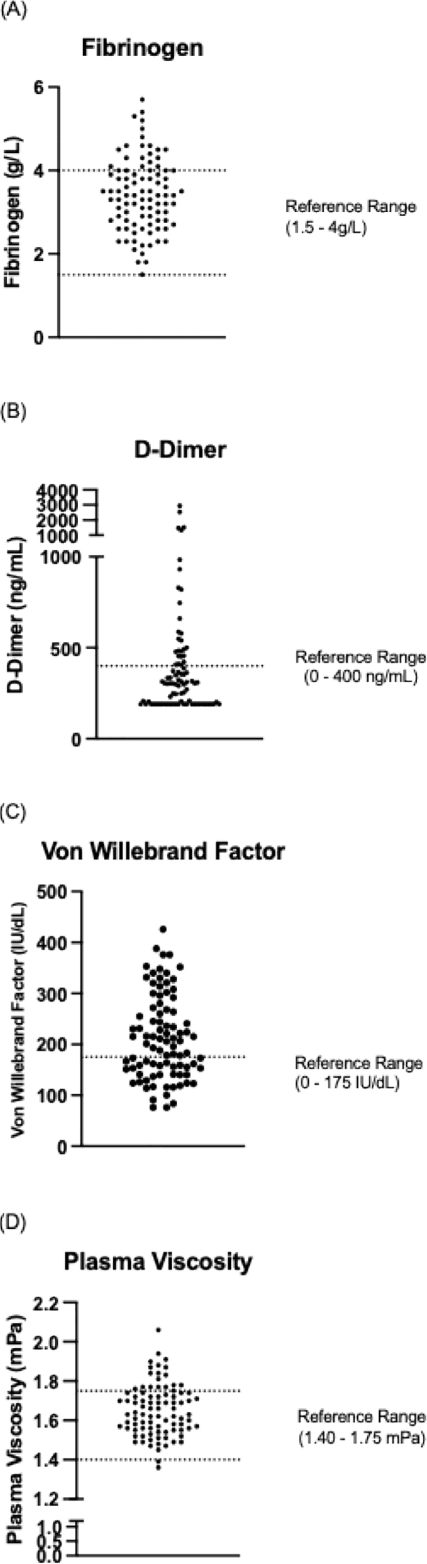
Distribution of **(A)** fibrinogen, **(B)** D-Dimer, **(C)** von Willebrand Factor and **(D)** plasma viscosity in the CVID patient cohort relative to reference ranges (dotted horizontal lines).

Abnormal coagulation parameters were common: elevated fibrinogen was observed in 23.3% (n=20), D-dimer in 31.4% (n=27), von Willebrand factor (vWF) in 57% (n=49), and plasma viscosity in 20.9% (n=18) (**Figure 1**). Additionally, 7% (n=6) tested positive for lupus anticoagulant, all of whom were receiving immunoglobulin replacement therapy at the time of testing. The median vWF level was elevated at 198.5 IU/dL (reference range: 45–175 IU/dL).

Inflammatory markers were commonly elevated: 22.1% (n=19) had increased CRP, and 59.3% (n=51) had elevated ESR.

The mean total cholesterol level was 4.49 mmol/L, with a mean LDL of 2.44 mmol/L, HDL of 1.39 mmol/L and triglycerides of 1.49 mmol/L. The mean HbA1c was 36.1 mmol/mol. Mean systolic and diastolic blood pressures were 124 mmHg and 75.7 mmHg, respectively. 14 patients were receiving treatment for hyperlipidaemia, and 22 were on antihypertensive therapy.

### A substantial number of patients have surrogate markers of cardiovascular disease or dyslipidaemia on imaging

Thoracic CT imaging was available for 92 patients. Coronary artery calcification was identified in 37% (n=34), with 20.7% (n=19) classified as mild, 7.6% (n=7) as moderate, and 6.5% (n=6) as severe. Two patients (2.2%) had coronary stents *in situ*. The remaining 63% (n=58) showed no evidence of coronary artery calcification. Among the 34 patients with calcification, 82.4% (n=28) had no prior history of cardiovascular disease, indicating subclinical atherosclerosis. Of these 28 patients, 35.7% (n=10) had moderate to severe calcification.

FibroScan CAP results were available for 60 patients. Hepatic steatosis was present in 30% (n=18), with 8.3% (n=5) classified as mild, 6.7% (n=4) as moderate, and 15% (n=9) as severe.

Pulse wave velocity measurements were available for 31 patients. 38.7% (n=12) patients had an aPWV <6 m/s, 54.8% (n=17) patients had an intermediate aPWV of 6–10 m/s, and 6.5% (n=2) had an aPWV of >10 m/s.

### CVID patients with coronary artery calcification have higher levels of coagulation markers

A total of 79 patients with thoracic CT imaging and complete blood data were included in this comparison.

Patients with coronary artery calcification on CT scan were significantly older (mean 65 years vs 45.9 years), and demonstrated higher rates of self-reported coronary artery disease (16.7% vs 0%, p=0.003), hypertension (50% vs 16.7%, p=0.001), hyperlipidaemia (66.7% vs 34.7%, p=0.006), diabetes or prediabetes (33.3% vs 6.1%, p=0.002), and chronic kidney disease (16.7% vs 0%, p=0.003) compared to those without calcification (**Table 3**).

**Table 3:** Comparison of demographics, cardiovascular disease, cardiovascular risk factors, blood markers of endothelial dysfunction and immunology blood markers in patients with CT evidence of coronary artery calcification and without coronary artery calcification.

Risk factors	No coronary artery calcification (n =49)	Coronary artery calcification (n=30)	*P* value
Age (years)	45.9 ± 14.2	65 ± 10.7	**<0.001** ^b^
Sex			
Male	18 (36.7%)	16 (53.3%)	0.148^a^
Female	31 (63.3%)	14 (46.7%)	
Ethnicity			
White	47 (95.9%)	29 (96.7%)	0.866^a^
Cardiovascular disease			
Coronary Artery Disease	0 (0%)	5 (16.7%)	0.003^a^
Cerebrovascular Accident	0 (0%)	1 (3.3%)	0.198^a^
Peripheral Vascular Disease	1 (2%)	0 (0%)	0.431^a^
Cardiovascular risk factors			
Hypertension	8 (16.7%)	15 (50.0%)	**0.001** ^a^
Hyperlipidaemia	17 (34.7%)	20 (66.7%)	**0.006** ^a^
Diabetes/Prediabetes	3 (6.1%)	10 (33.3%)	**0.002** ^a^
Chronic Kidney Disease	0 (0%)	5 (16.7%)	**0.003** ^a^
Smoking history	10 (20.4%)	5 (16.7%)	0.681^a^
Cardiovascular risk indicators			
Total Cholesterol (mmol/L)	4.58 ± 0.99	4.63 ± 1.21	0.855^b^
LDL (mmol/L)	2.45 ± 0.85	2.57 ± 0.99	0.573^b^
HDL (mmol/L)	1.47 ± 0.57	1.29 ± 0.40	0.141^b^
Triglycerides (mmol/L)	1.46 ± 0.83	1.73 ± 0.93	0.186^b^
Hba1C (mmol/mol)	34.5 ± 4.1	40.3 ± 16.3	**0.020** ^b^
Systolic Blood Pressure (mmHg)	123.1 ± 16.0	131.4 ± 18.7	0.054^b^
Diastolic Blood Pressure (mmHg)	76.6 ± 10.5	77.8 ± 8.1	0.624^b^
Blood markers of endothelial dysfunction			
Platelets (x10^9^/L)	198.3 ± 78.1	170.1 ± 73.3	0.115^b^
Fibrinogen (g/L)	3.30 ± 0.70	3.57 ± 1.04	0.174^b^
D-Dimer (ng/mL)	271.0 (190.0 – 389.0)	370.5 (208.8 – 599.5)	**0.011** ^c^
Von Willebrand Factor (IU/dL)	167.0 (140.0 – 233.0)	227.5 (199.0 – 322.8)	**0.001** ^c^
Lupus Anticoagulant	4 (8.2%)	2 (6.7%)	0.808^a^
Plasma Viscosity (mPa)	1.67 ± 0.12	1.65 ± 0.15	0.617^b^
CRP (mg/L)	3.0 (1.0 - 5.6)	2.6 (1.0 - 4.6)	0.592^c^
ESR (mm/hr)	8.0 (5.0 - 15.5)	8.5 (2.0 - 20.3)	0.579^c^
Immunology parameters			
IgA (g/L)	0.1 (0.1 – 0.1)	0.1 (0.1 – 0.3)	0.117^c^
IgM (g/L)	0.1 (0.1 – 0.5)	0.1 (0.1 – 0.5)	0.539^c^
IgG (g/L)	10.5 (8.4 – 11.8)	9.7 (8.0 – 11.5)	0.437^c^
Switched memory B cells (% of B cells)	2.23 (1.25-4.76)	4.44 (1.40-7.93)	0.113^c^
CD21^low^ B cells (% of B cells)	11.61 (6.75 - 27.00)	10.54 (5.76 - 27.00)	0.688^c^
**Arterial Pulse Wave Velocity (m/s)**	6.00 ± 1.14	8.20 ± 2.87	**0.006** ^b^
**FibroScan CAP (dB/m)**	213.0 (174.5 – 244.0)	222.0 (141.0 – 274.0)	0.783^c^

a Chi-squared test

b Student’s t-test

c Mann-Whitney U test

low-density lipoprotein, (LDL); high-density lipoprotein, (HDL); Hba1C, glycated haemoglobin; CRP, C-reactive protein; ESR, erythrocyte sedimentation rate; immunoglobulin A (IgA); immunoglobulin M (IgM); immunoglobulin G (IgG)

Findings are presented as mean ± standard deviation for variables with a normal distribution, and as median with interquartile range (25th–75th percentile) for non-normally distributed variables.Bold values indicate statistical significance (p < 0.05).

Mean cholesterol levels and blood pressure trended higher in patients with coronary artery calcification, although these differences did not reach statistical significance. HbA1c was significantly elevated in patients with coronary artery calcification (34.5 mmol/mol vs 40.3 mmol/mol, p=0.020).

**Figure 2:**
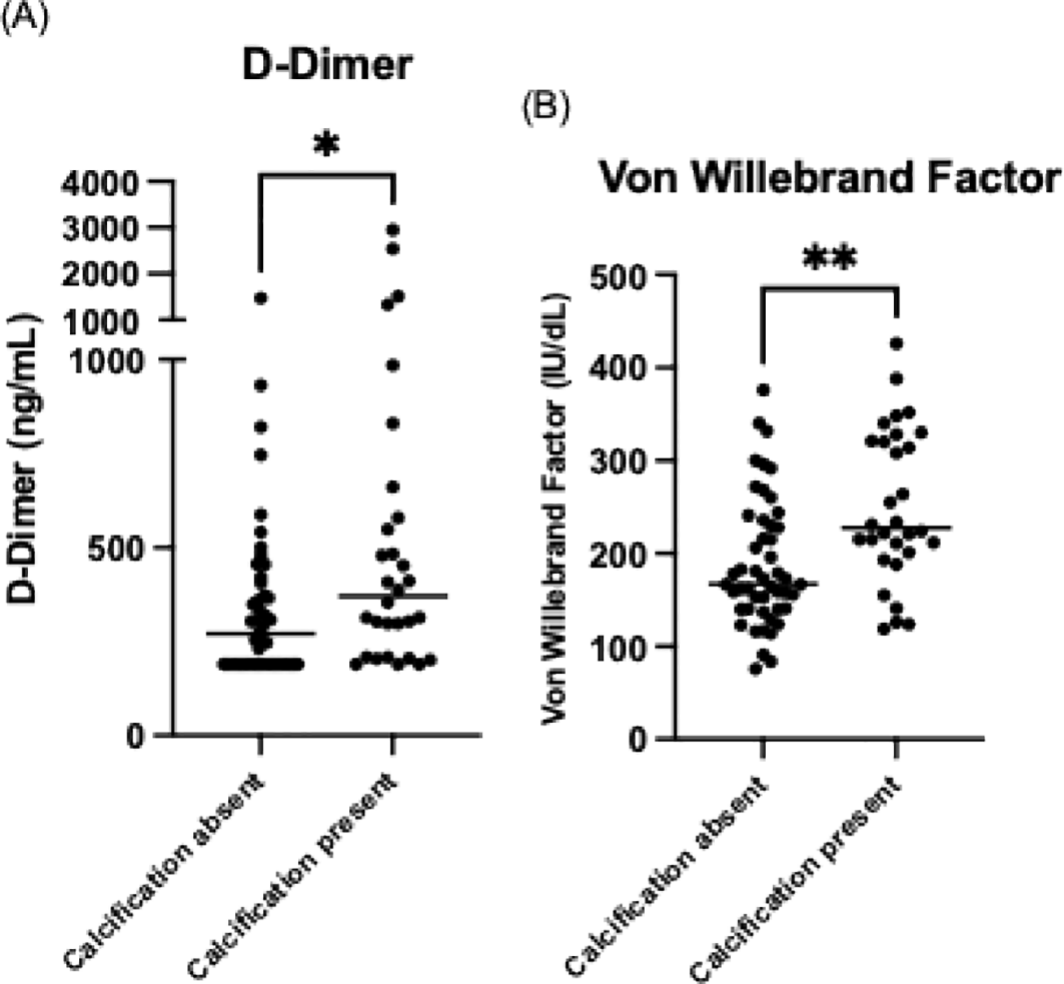
Comparison of **(A)** D-Dimer and **(B)** von Willebrand factor levels in CVID patients with and without coronary artery calcification on CT imaging. The horizontal lines represent the median values for D-dimer and von Willebrand factor. *p < 0.05, **p < 0.01.

Among the markers of hypercoagulability, von Willebrand factor levels were significantly higher in patients with calcification (227.5 IU/dL vs 167 IU/dL, p=0.001), as were D-dimer levels (370.5 ng/mL vs 271 ng/mL, p=0.011) (**Figure 2**). No significant differences were observed in platelet count, lupus anticoagulant, fibrinogen, or plasma viscosity. Inflammatory markers, including CRP and ESR, also showed no statistically significant differences between groups.

With respect to other surrogate measures of cardiovascular disease, arterial pulse wave velocity (PWV) was significantly higher in patients with coronary artery calcification (8.2 m/s vs 6 m/s, p=0.006). However, liver CAP values did not differ significantly between groups.

### Subgroup analysis of coronary artery calcification within the inflammatory phenotype

Subgroup analyses were performed to evaluate the association between non-infectious CVID phenotypes (GLILD, enteropathy, and liver disease) and the presence of coronary artery calcification. Among patients with an inflammatory phenotype, 62 had available CT imaging. Within this group, 16 patients (25.8%) had GLILD, 32 (51.6%) had enteropathy, and 33 (53.2%) had liver disease. Evidence of CAC was present in 6 patients (37.5%) with GLILD, 10 (31.3%) with enteropathy, and 13 (39.4%) with liver disease. No statistically significant differences in CAC prevalence were observed between these subgroups (GLILD vs no GLILD, p=0.929; enteropathy vs no enteropathy, p=0.325; liver disease vs no liver disease, p=0.690).

### CVID patients with inflammatory phenotype have elevated markers of coagulation

There were 34 patients (33.7%) with an infection only phenotype and 67 patients (66.3%) with an inflammatory phenotype. The mean age was 51.4 years in the infection only group and 52.5 years in the inflammatory group. The sex distribution was 38.2% male (n=13) in the infection only group and 49.3% male (n=33) in the inflammatory group (**Table 4**).

**Table 4:** Comparison of demographics, cardiovascular disease, cardiovascular risk factors, blood markers of endothelial dysfunction and immunology blood markers in patients with infection only phenotype and inflammatory phenotype.

Risk factors	Infection only (n =34)	Inflammatory (n=67)	*p* value
**Age (years)**	51.4 ± 16.3	52.5 ± 15.9	0.729^b^
Sex			
Male	13 (38.2%)	33 (49.3%)	0.293^a^
Female	21 (61.8%)	34 (50.7%)	
Ethnicity			
White	31 (91.2%)	62 (92.5%)	0.811^a^
Cardiovascular disease			
Coronary Artery Disease	0 (0%)	5 (7.5%)	0.102^a^
Cerebrovascular Accident	1 (2.9%)	1 (1.5%)	0.621^a^
Peripheral Vascular Disease	1 (2.9%)	0 (0%)	0.158^a^
Cardiovascular risk factors			
Hypertension	10 (29.4%)	14 (20.9%)	0.342^a^
Hyperlipidaemia	16 (47.1%)	23 (34.3%)	0.214^a^
Diabetes/Prediabetes	3 (8.8%)	12 (17.9%)	0.225^a^
Chronic Kidney Disease	0 (0%)	7 (10.5%)	**0.050** ^a^
Smoking history	4 (11.8%)	13 (19.4%)	0.332^a^
Cardiovascular risk indicators			
Total Cholesterol (mmol/L)	4.65 ± 0.90	4.42 ± 1.15	0.338^b^
LDL (mmol/L)	2.50 ± 0.80	2.41 ± 0.95	0.649^b^
HDL (mmol/L)	1.43 ± 0.47	1.37 ± 0.54	0.567^b^
Triglycerides (mmol/L)	1.61 ± 0.93	1.43 ± 0.77	0.326^b^
Hba1C (mmol/mol)	34.8 ± 3.8	36.8 ± 12.2	0.374^b^
Systolic Blood Pressure (mmHg)	124.7 ± 17.2	123.7 ± 17.5	0.815^b^
Diastolic Blood Pressure (mmHg)	76.2 ± 10.4	75.5 ± 9.1	0.728^b^
Blood markers of endothelial dysfunction			
Platelets (x10^9^/L)	240.1 ± 59.9	159.2 ± 73.6	**<0.001** ^b^
Fibrinogen (g/L)	3.43 ± 0.75	3.41 ± 0.92	0.919^b^
D-Dimer (ng/mL)	205 (190 – 406)	314 (208 – 481)	**0.043** ^c^
Von Willebrand Factor (IU/dL)	163.0 (124.5 – 206.8)	224.0 (159.0 – 305.0)	**<0.001** ^c^
Lupus Anticoagulant	3 (11.1%)	3 (5.1%)	0.309^a^
Plasma Viscosity (mPa)	1.67 ± 0.13	1.64 ± 0.13	0.369^b^
CRP (mg/L)	2.0 (1.0 - 5.0)	2.7 (1.0 – 5.3)	0.292^c^
ESR (mm/hr)	6.0 (2.0 – 12.0)	8.0 (2.0 - 16.3)	0.387^c^
Immunology parameters			
IgA (g/L)	0.1 (0.1 – 0.4)	0.1 (0.1 – 0.1)	0.014^c^
IgM (g/L)	0.2 (0.1 – 0.5)	0.1 (0.1 – 0.4)	0.195^c^
IgG (g/L)	9.7 (8.0 – 12.1)	10.3 (8.7 – 11.7)	0.646^c^
Switched memory B cells (% of B cells)	5.57 (2.37-13.60)	1.93 (0.92-4.35)	**<0.001** ^c^
CD21^low^ B cells (% of B cells)	6.81 (4.71 – 11.96)	22.70 (9.91 – 37.13)	**<0.001** ^c^
Arterial Pulse Wave Velocity (m/s)	6.86 ± 2.93	6.46 ± 1.42	0.615^b^
FibroScan CAP (dB/m)	224.0 (208.0 – 297.0)	205.0 (174.5 – 258.5)	0.115^c^

a Chi-squared test

b Student’s t-test

c Mann-Whitney U test

immunoglobulin A, (IgA); immunoglobulin M, (IgM); immunoglobulin G, (IgG)

Findings are presented as mean ± standard deviation for variables with a normal distribution, and as median with interquartile range (25th–75th percentile) for non-normally distributed variables.Bold values indicate statistical significance (p < 0.05).

**Figure 3:**
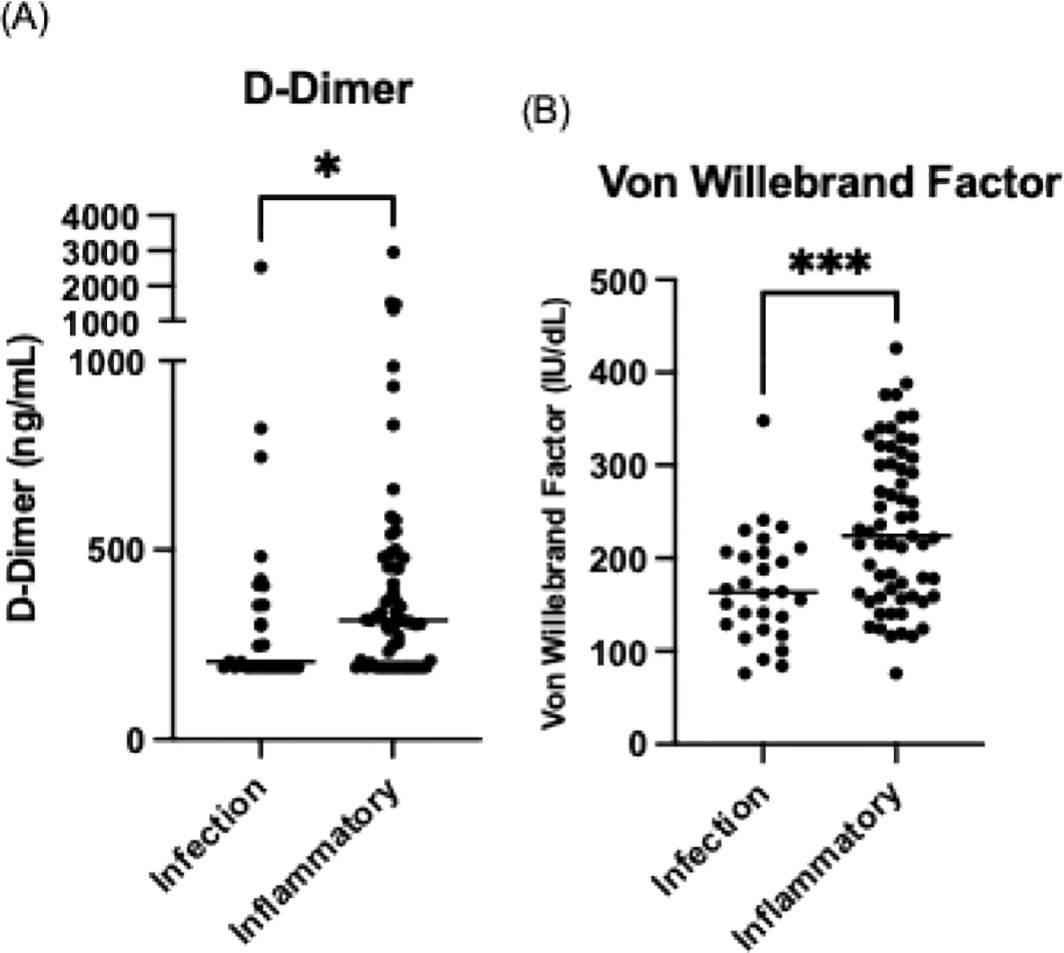
Comparison of **(A)** D-Dimer and **(B)** von Willebrand Factor levels in CVID patients with infection-only versus inflammatory phenotypes. The horizontal line represents the median value. *p < 0.05, ***p < 0.001.

The inflammatory phenotype group had a higher prevalence of chronic kidney disease (10.5% [n=7] vs 0% [n=0], p=0.05). Known coronary artery disease was also more common in the inflammatory group (7.5% [n=5] vs 0% [n=0], p = 0.102), although this did not reach statistical significance. The inflammatory phenotype was associated with significantly lower median switched memory B cells (1.93% vs 5.57%, p<0.001) and higher median CD21^low^ B-cell proportions (22.7% vs 6.81%, p<0.001). Mean platelet counts were lower in the inflammatory group (159.2 vs 240.1 ×10^9^/L, p<0.001). Additionally, the median D-dimer level was significantly elevated in this group (314 ng/L vs 205 ng/L, p=0.043), as was the median von Willebrand factor level (224 IU/dL vs 163 IU/dL, p<0.001) (**Figure 3**).

**Figure 4:**
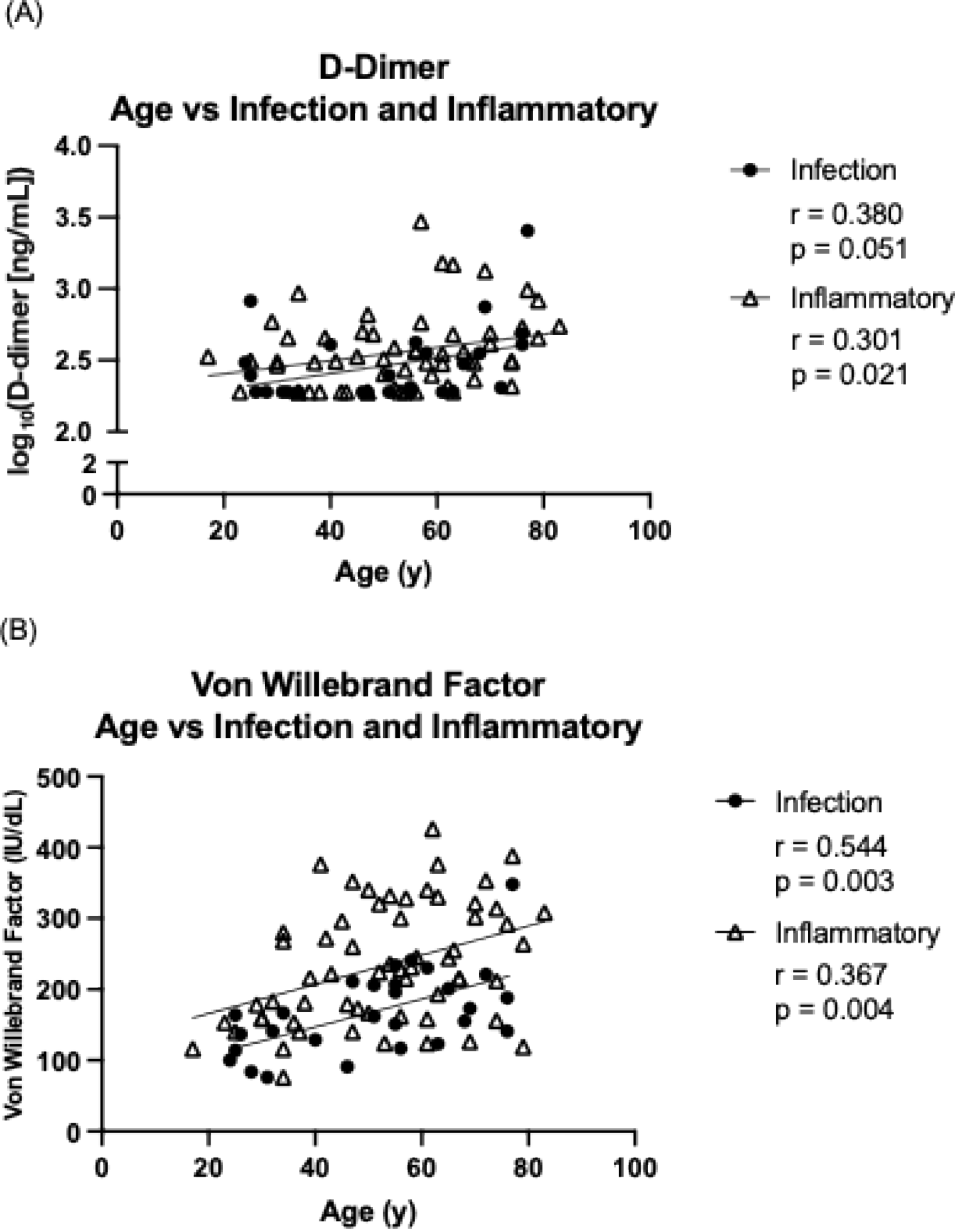
Scatter plots showing the relationships between **(A)** age and log_10_ (D-dimer), and **(B)** age and von Willebrand Factor (vWF) in CVID patients. A linear trend line is shown for visualisation purposes only. Spearman’s rank correlation was used for statistical analysis due to non-parametric data distribution.

The association between age and biomarker levels (D-dimer vWF) was evaluated separately within the infection-only and inflammatory CVID phenotype groups. Spearman’s rank correlation revealed a positive relationship between age and both biomarkers in each subgroup. For D-dimer, a moderate correlation was observed in the infection-only group (r=0.380, p=0.051), approaching statistical significance, and a statistically significant correlation was noted in the inflammatory group (r=0.301, p=0.021). For vWF, stronger positive correlations were identified in both the infection-only (r=0.544, p=0.003) and inflammatory (r=0.367, p=0.004) groups. Notably, log_10_ D-Dimer and vWF levels were consistently higher across all age ranges in the inflammatory phenotype group (**Figures 4A, B**).

## Discussion

The key findings of our study are that patients with CVID have a higher burden of cardiovascular risk factors and subclinical atherosclerosis compared with controls. Elevated biomarkers of endothelial dysfunction, particularly D-dimer and vWF, were associated with coronary artery calcification and were more pronounced in patients with an inflammatory phenotype. Biomarker abnormalities persisted even after accounting for age, suggesting that additional disease related mechanisms such as immune dysregulation and systemic inflammation contribute to vascular injury and thrombotic risk. Prioritising infection prevention alone in CVID may overlook broader health needs, and a more holistic approach that integrates cardiovascular screening and prevention is essential for optimising long term outcomes.

To the best of our knowledge, this is the first study to provide a comprehensive evaluation of cardiovascular risk integrating biomarkers of endothelial dysfunction and imaging surrogates of atherosclerosis in patients with CVID. Coronary artery calcification was observed in 37% of patients on thoracic CT imaging, with severity ranging from mild to severe. Notably, the majority of these patients (82.4%) were asymptomatic, despite nearly one-third exhibiting moderate to severe coronary artery calcification. Hepatic steatosis, another marker of cardiometabolic risk, was present in approximately 30% of patients. Increased pulse wave velocity (aPWV >10 m/s), a measure of arterial stiffness and independent predictor of cardiovascular events, was observed in 6.5%, while 54.8% had intermediate aPWV (6–10 m/s) indicating early vascular changes (22, 25, 26). As expected, CVID patients with coronary artery calcification demonstrated significantly higher rates of traditional cardiovascular risk factors and comorbidities, including hypertension, hyperlipidaemia, diabetes, chronic kidney disease, and in some cases, established coronary artery disease, compared to CVID patients without calcification.

The cardiovascular risk factors identified in our study are comparable to those reported in other cohorts with autoimmune rheumatic diseases. Compared with the study by Bolla et al. (27), our cohort had a lower prevalence of hypertension (23.8% vs 35.6%) but higher rates of hyperlipidaemia (38.6% vs 19.8%), although our cohort was older (median age 55 vs 43 years). Similarly, compared with the autoimmune rheumatic disease cohort described by Gumber et al. (28), our cohort showed similar rates of hypertension (23.8% vs 25.6%) but higher rates of type 2 diabetes (14.9% vs 7.7%), despite being younger (median age 55 vs 63 years). Consistent with findings in autoimmune rheumatic diseases and HIV (29, 30), coronary artery calcification was observed in a substantial proportion of our cohort. Notably, the majority (82.4%) had no prior history of cardiovascular disease. Hansen et al. reported that patients with rheumatoid arthritis exhibit a higher prevalence of asymptomatic coronary artery disease, with greater mean coronary calcium scores, more frequent multivessel involvement, and a higher proportion of high-risk plaques compared with controls (29). Likewise, Karady et al. found a higher prevalence of coronary plaque on CT in asymptomatic people living with HIV than in HIV negative controls (30). Compared with the general population, coronary artery calcification prevalence was higher in our cohort (37%) than the 29.9% reported among individuals aged 50–54 years in the study by Bergström et al. (31).

Our findings align with emerging literature linking CVID to premature atherosclerosis. Mattila et al. reported that CVID patients have more than twice the odds of developing coronary heart disease and peripheral vascular disease compared to matched controls (6). In that study, hypertension was more prevalent among CVID patients. Similarly, our cohort of CVID patients with coronary artery calcification demonstrated increased rates of hypertension and hyperlipidaemia. However, despite the frequent presence of cardiovascular risk factors, few cardiovascular diseases were reported in our study, in contrast to Mattila et al. This discrepancy may reflect earlier clinical intervention, limited statistical power to detect cardiovascular disease due to sample size, or a potential protective effect of immunoglobulin therapy. Higher IgG levels have been associated with reduced cardiovascular disease (32); notably, IgG trough levels trended higher in patients without coronary artery calcification in our study, although this difference did not reach statistical significance.

One of the key findings in our study was the association between elevated vWF and D-dimer levels with the presence of coronary artery calcification. Mattila et al. concluded that CVID itself is an independent risk factor for atherosclerosis, suggesting that underlying immune dysregulation accelerates vascular damage beyond the contribution of traditional risk factors (6). Elevated D-dimer, a fibrin degradation product, is a marker of a hypercoagulable state and is independently associated with future coronary events and mortality (33, 34). Similarly, elevated vWF similarly reflects endothelial activation and is linked to atherothrombosis, whereas lower vWF levels have been associated with reduced cardiovascular risk (35, 36). In our CVID cohort, these abnormalities were present even in younger patients with an inflammatory phenotype, potentially suggesting a heightened baseline vascular risk.

In our study, both D-dimer and vWF levels were observed to increase with age among CVID patients. This aligns with the literature that cardiovascular risk factors and D-dimer rise with age due to increased fibrin turnover and subclinical vascular injury (37–39). This rise is thought to reflect greater fibrin turnover, endothelial dysfunction, and the accumulation of metabolic and inflammatory comorbidities such as dyslipidaemia, anaemia, and obesity (39). Similarly, ageing is associated with increased vWF levels (40, 41). Despite this association, our findings suggest that age alone does not fully account for the observed elevations in vWF and D-dimer. Across all age ranges, CVID patients with an inflammatory phenotype had consistently higher levels of both markers compared to those with an infection-only phenotype. Correlation analyses further support this: age correlated more strongly with vWF and D-dimer in the infection-only group, while in the inflammatory group, the correlation was weaker. This disparity implies that, in the inflammatory phenotype, additional disease-related factors beyond aging may contribute to the elevated levels of these biomarkers, such as chronic immune activation or underlying systemic inflammation. We also note that the elevations in D-dimer and vWF were frequently beyond the upper limit of the normal range. In exploratory subgroup analyses, no statistically significant differences in coronary artery calcification prevalence were observed between patients with specific non-infectious CVID complications, including GLILD, enteropathy or liver disease. This suggests that cardiovascular risk in CVID may be driven by shared mechanisms related to chronic systemic inflammation and immune dysregulation rather than by individual inflammatory manifestations alone. However, subgroup sizes were small and overlapping phenotypes were common, limiting statistical power to detect modest differences.

This hypothesis is supported by previous findings in autoimmune diseases, where chronic inflammation contributes significantly to endothelial injury and cardiovascular risk (2). Inflammation is known to impair HDL synthesis via reduced expression of Apo-A1, LCAT, and ABCA1, while low HDL may itself perpetuate inflammation (8, 9). Antioxidant markers such as selenium and glutathione peroxidase are also reduced in CVID (15). In our cohort, CVID patients with an inflammatory phenotype demonstrated higher CD21^low^ B cell percentages, consistent with prior literature linking this phenotype to immune dysregulation (19). Interestingly, despite elevated coagulation markers, patients with an inflammatory phenotype did not show higher rates of cardiovascular disease or coronary artery calcification on imaging. One possible explanation is that this group was significantly younger than those with radiological evidence of coronary artery calcification and may develop cardiovascular risk factors and disease later in life. The widespread use of polyvalent IgG replacement therapy in CVID has significantly reduced infectious complications and improved long term prognosis. Beyond antimicrobial protection, immunoglobulin therapy may exert a protective effect against cardiovascular risk, potentially mitigating endothelial dysfunction. Immunoglobulin is known to neutralise autoantibodies, modulate cytokine production, and regulate immune cell function (42). Improvements in flow-mediated dilatation following intravenous immunoglobulin (IVIG) infusion have been reported in patients with CVID, and human immunoglobulin has been shown to stimulate nitric oxide production from endothelial cells *in vitro* (10). IVIG has also been associated with upregulation of FcγRIIB expression, contributing to suppression of inflammation (43). However, we observed that inflammatory markers remained elevated in our inflammatory cohort despite adequate IgG replacement, suggesting persistent immune dysregulation beyond infection control.

Currently, there are no established guidelines for cardiovascular screening in patients with CVID. Given that our cardiovascular findings are comparable to those observed in HIV and autoimmune disease cohorts, it may be reasonable to extrapolate these recommendations. Current guidelines, including those from the European Society of Cardiology (ESC), the American Heart Association (AHA), the European Alliance of Associations for Rheumatology (EULAR) recommend aggressive modification of conventional risk factors and consideration of imaging modalities such as coronary calcium scoring or carotid ultrasound to detect subclinical atherosclerosis (44–47). Traditional cardiovascular risk calculators such as Framingham Risk Score, Atherosclerotic Cardiovascular Disease Risk Calculator (ASCVD), Systematic Coronary Risk Evaluation 2 (SCORE2) tend to underestimate risk in patients with chronic inflammation, as they fail to account for immune dysregulation or treatment related effects (46, 47). Consequently, the QRISK3 model includes rheumatoid arthritis and systemic lupus erythematosus to better capture inflammation associated cardiovascular risk (48). The AHA and The European AIDS Clinical Society (EACS) guidelines recognise HIV as a high risk condition warranting earlier and more intensive risk modification (45, 49). Similarly, the EULAR recommendations suggest applying a 1.5 - 2.0 multiplication factor to standard risk scores or using imaging-based assessment (46). Given our findings, it is likely that existing risk calculators will also underestimate cardiovascular risk in patients with CVID, particularly those with an inflammatory phenotype. In addition, traditional cardiovascular risk calculators such as SCORE2 and the ASCVD risk estimator are designed for the general population aged 40 years and older, limiting their applicability in younger patients with CVID (46, 47).

Traditional cardiovascular risk calculators highlight the limitations of conventional risk algorithms in immune mediated conditions and support the need for disease specific or inflammation adjusted cardiovascular screening strategies. Our study demonstrated elevated vWF and D-dimer levels in patients with coronary artery calcification and an inflammatory phenotype. Previous studies have shown D-dimer to be predictive of cardiovascular disease (33, 34, 50), and VWF to be an independent prognostic biomarker for coronary artery disease and major adverse cardiovascular events (35, 36, 51, 52). In individuals living with HIV, Duprez et al. reported that IL-6, hsCRP and D-dimer were independently associated with cardiovascular risk (53), while Kanmogne et al. demonstrated persistent endothelial activation and elevated VWF despite antiretroviral therapy, linking HIV related inflammation to endothelial dysfunction and thrombosis (54). Although neither biomarker is currently included in clinical guidelines for cardiovascular screening, their strong prognostic associations in inflammatory populations highlight potential utility for identifying individuals at elevated cardiovascular risk, an approach that could also be useful for CVID.

There are some limitations to our study. Cardiovascular risk factors and disease were self-reported by controls, which may be subject to recall bias, whereas we measured some of these parameters directly in patients. Laboratory and imaging data were obtained from routine clinical care and research assessments, and therefore, not all variables were available for every participant. Coagulation and immunology blood tests were performed only in CVID patients, with no matched data available for controls, limiting direct comparison. Additionally, thoracic CT imaging, aPWV, and FibroScan (CAP) were not obtained for the control group. Single time-point measurements were used for immunoglobulins and coagulation markers, rather than serial or median values over time. Some coagulation markers, such as D-Dimer, are acute-phase reactants and may be influenced by transient inflammatory states. To mitigate this, Mann–Whitney U test was applied for non-parametric data analysis. Coronary artery calcification assessment was qualitative rather than a formal Agatston score. However, semi-quantitative assessment of coronary artery calcification on non-gated thoracic CT has been shown to be reliable and is endorsed by imaging societies (24). This cohort may be subject to selection bias, with a higher proportion of patients exhibiting an inflammatory phenotype than an infection only phenotype, likely due to referral to a major tertiary immunology centre that manages complex CVID cases in the UK. The study population was skewed toward individuals identifying as white, which may affect generalisability and potentially reflect better healthcare access, resulting in fewer reported cardiovascular diseases. Nevertheless, the use of household members as controls mitigated some confounders by better matching for environmental and lifestyle factors, although individual lifestyle differences may still exist within the same household. In addition, owing to the lack of laboratory and blood pressure data for healthy controls, the use of traditional cardiovascular risk calculators as a comparative measure was not feasible. Although no statistically significant differences in coronary artery calcification were observed among inflammatory phenotype subgroups, the small sample sizes likely limited statistical power, and this warrants further evaluation in future studies. An additional strength of this study lies in the integration of objective imaging findings with a comprehensive biomarker panel and detailed clinical history.

## Conclusion

This study provides the first comprehensive assessment of endothelial dysfunction and coagulation biomarkers alongside radiographic indicators of atherosclerosis in patients with CVID. Our findings reveal a significant burden of cardiovascular risk factors and vascular abnormalities in this population, even in the absence of overt cardiovascular disease. Elevated vWF and D-dimer levels were particularly prominent in patients with coronary artery calcification and in those with an inflammatory CVID phenotype. These associations persisted across age groups, suggesting that chronic immune dysregulation and systemic inflammation may contribute to vascular injury beyond the effects of ageing alone. As individuals with CVID live longer due to advances in immunoglobulin replacement and clinical care, attention must shift toward identifying and managing non-infectious comorbidities, including cardiovascular risk. Further longitudinal studies are needed to determine whether biomarker abnormalities predict clinical cardiovascular outcomes in this patient population.

## Data Availability

The original contributions presented in the study are included in the article/**Supplementary Material**. Further inquiries can be directed to the corresponding author.
