# Supplementary material for: Risk factors and surrogate indicators for cardiovascular disease are prevalent in Common Variable Immunodeficiency and associate with inflammatory phenotype
Source: Front Immunol. 2026 Mar 2;17:1756049. doi: 10.3389/fimmu.2026.1756049 (PMC12989573; doi:10.3389/fimmu.2026.1756049)
Supplement: Supplementary file 1 [file Table1.docx]

**Supplementary Materials**

|  | **CVID (n= 46)** | **Control (n= 56)** | ***p* value** |
| --- | --- | --- | --- |
| **Age (years)** | 52.2 ±17.2 | 51.6 ±16.5 | 0.883 |
| **Sex** |  |  |  |
| Male | 23 (50%) | 27 (48.2%) | 0.858 |
| Female | 23 (50%) | 29 (51.8%) |  |
| **Ethnicity** |  |  |  |
| White | 42 (91.3%) | 51 (91.1%) | 0.967 |
| Non-white | 4 (8.7%) | 5 (8.9%) |  |
| **Cardiovascular risk factors (self-reported or established via diagnostic tests)** |  |  |  |
| Hypertension | 6 (13%) | 14 (25%) | 0.130 |
| Hyperlipidaemia | 17 (37%) | 13 (23.2%) | 0.130 |
| Diabetes/Prediabetes | 5 (10.9%) | 8 (14.3%) | 0.607 |
| Chronic Kidney Disease | 3 (6.5%) | 0 (0%) | 0.052 |
| Smoking history | 9 (19.6%) | 5 (8.9%) | 0.120 |
| **Cardiovascular Events** |  |  |  |
| Coronary Artery Disease | 3 (6.5%) | 4 (7.1%) | 0.902 |
| Cerebrovascular Accident | 1 (2.2%) | 3 (5.4%) | 0.410 |
| Peripheral Vascular Disease | 1 (2.2%) | 1 (1.8%) | 0.888 |

Supplementary Table 1: Comparison of CVID cohort versus controls, restricted to those patients with matched household controls

Abbreviations: CVID, common variable immunodeficiency disorder
